# Rao and Wald Tests for Nonhomogeneous Scenarios

**DOI:** 10.3390/s120404730

**Published:** 2012-04-12

**Authors:** Chengpeng Hao, Danilo Orlando, Chaohuan Hou

**Affiliations:** 1 The State Key Laboratory of Acoustics, Institute of Acoustics, Chinese Academy of Sciences, Beijing 100190, China; 2 Elettronica S.P.A., Via Tiburtina Valeria, Rome 00131, Italy

**Keywords:** nonhomogeneous scenarios, Rao test, Wald test, mismatch

## Abstract

In this paper, we focus on the design of adaptive receivers for nonhomogeneous scenarios. More precisely, at the design stage we assume a mismatch between the covariance matrix of the noise in the cell under test and that of secondary data. Under the above assumption, we show that the Wald test is the adaptive matched filter, while the Rao test coincides with the receiver obtained by using the Rao test design criterion in homogeneous environment, hence providing a theoretical explanation of the enhanced selectivity of this receiver.

## 1. Introduction

In recent years adaptive detection of targets embedded in Gaussian disturbance with unknown spectral properties has received an increased attention in the signal processing community. In the seminal paper by Kelly [1], the generalized likelihood ratio test (GLRT) is used to design an adaptive decision scheme capable of detecting a signal known up to a scaling factor in presence of Gaussian disturbance with unknown covariance matrix. Moreover, it is assumed that a set of secondary data free of signal components, but sharing the same spectral properties of the noise in the cell under test (CUT), is available (homogeneous environment). In [2] the authors derive the so-called adaptive matched filter (AMF), whose design relies on the so-called two-step GLRT-based design procedure. In fact, its design is split into two steps: first a non-adaptive GLRT for known covariance matrix is derived; then, the fully-adaptive version of the GLRT is obtained by replacing the unknown matrix with a proper estimate. Other design criteria have been investigated as an alternative to the GLRT over the years. In particular, in [3] a novel derivation of the AMF is proposed by resorting to the Wald test design criterion for the homogeneous environment. In [4], the Rao test is used to derive a detector (referred to in the following as H-RAO) that exhibits enhanced rejection capabilities of mismatched signals [5–7].

However, the homogeneous environment is an assumption that might not be met in realistic situations: see, for example, ([8], and references therein). A slightly general model assumes that the covariance matrix of the CUT and that of secondary data coincide only up to a scale factor. This scenario is referred to as partially-homogeneous environment and has been firstly proposed in [9], where the authors apply the GLRT to derive a fully-adaptive detector, referred to as the adaptive coherence estimator (ACE) but also as adaptive normalized matched filter [10]. Interestingly, in [11] it is proven that Rao and Wald tests for partially-homogeneous environment coincide with the ACE.

Another model of interest assumes that CUT and secondary data share the same covariance matrix of the thermal noise plus clutter, but, in addition, the CUT contains a noise-like interferer [12]. Such a covariance mismatch can lead to receivers that are less inclined to reveal signals that produce steering vectors different from the nominal one (selective receivers) [13]. In [12] the authors show that the GLRT for this problem is the ACE, providing, as a byproduct, a theoretical explanation of the good rejection capabilities of such a receiver. The above model is modified in [14], where it is assumed that the noise-like interferer is orthogonal to the nominal steering vector in the whitened observation space. The goal is to induce the rejection of only those signals which are sufficiently far from the nominal steering vector. As a matter of fact, the GLRT for this constrained problem is Kelly's detector, which offers a good tradeoff between detection performance of mainlobe targets and rejection capabilities of sidelobe interferers [14].

In this work, we use the Wald test and the Rao test design criteria to solve the latter detection problem. In particular, we show that these design criteria yield the decision statistics that have been obtained for the homogeneous environment, *i.e.*, the Wald test is the AMF, while the Rao test coincides with the H-RAO. Moreover, the latter coincidence provides, as a byproduct, an alternative explanation of the excellent selectivity exhibited by the H-RAO in the homogeneous scenario; in fact, taking into account the noise-like interferer at the design stage can make it possible to reject the signal-plus-noise hypothesis as the degree of mismatch between the received signal and the postulated one increases. The interested reader is referred to ([5] and references therein) for the performance assessment of the above detectors in comparison to existing receivers in open literature.

The remainder of the paper is organized as follows: the next section is devoted to the problem formulation while Section III addresses detector designs. Finally, Section IV contains some concluding remarks and potential directions for future works.

## 2. Problem Formulation

Assume that a linear array formed by *N_a_* ∈ IN antennas senses the cell under test and that each antenna collects *N_t_* ∈ IN samples. Denote by *z* ∈ ℂ *^N^*^×^*^1^* the *N*-dimensional vector, with *N* = *N_a_N_t_*, containing returns from the CUT. We want to test whether or not *z* contains useful target echoes. As customary, we assume that a set of *K* ∈ IN secondary data, *z_k_* ∈ ℂ*^N^*^×1^, *k* = 1,…, *K, K* ≥ *N*, namely data free of signal components is available.

The detection problem at hand can be formulated as follows:

(1) $$\{\begin{array}{l} H_{0}:\{\begin{array}{cc} \text{z}=\text{n}, & \\ {\text{z}}_{k}={\text{n}}_{k}, & k=1,\ldots ,K \end{array}\\ H_{1}:\{\begin{array}{cc} \text{z}=\alpha \text{υ}+\text{n}, & \\ {\text{z}}_{k}={\text{n}}_{k}, & k=1,\ldots ,K \end{array} \end{array}$$

where

- ***υ*** ∈ ℂ*^N^*^×1^ is the nominal steering vector;
- ***n**_k_* ∈ ℂ*^N^*^×1^, *k* = 1,…, *K*, are independent and identically distributed complex normal random vectors with zero mean and unknown, positive definite covariance matrix ***M*** ∈ ℂ*^N^*^×^*^N^, i.e., ***n***_k_* ∼ 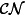
*_N_*(***0***, ***M***), *k* = 1,…, *K*, see ([5] and references therein);
- *n* ∼ 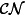
*_N_*(0, ***M*** + ***qq***^†^), where *q* ∈ℂ*^N^*^×1^ is due to a noise-like interferer and ^†^ denotes conjugate transpose. Moreover, we assume that ***q*** is orthogonal to the nominal steering vector in the whitened observation space, namely
  (2) $${\text{q}}^{†}{\text{M}}^{-1}\text{υ}=0$$
- *α* ∈ ℂ an unknown (deterministic) factor which accounts for both target and channel effects.

Notice that the probability density function (pdf) of *z* and ***Z*** ≡ [*z*_1_,…, *z_K_*] under *H*_1_ (and assuming that Equation (2) holds true) is given by

(3) $$f(z,\text{Z};\text{θ},H_{1})=\frac{\exp\left\{-\text{tr}\left[{\text{M}}^{-1}(\text{S}+(z-\alpha \upsilon ){\left(z-\alpha \upsilon \right)}^{†})\right]\right\}}{\pi^{N(K+1)}\det{\left(\text{M}\right)}^{K+1}(1+{\text{q}}^{†}{\text{M}}^{-1}\text{q})}\times \exp\left\{\frac{{\left|z^{†}{\text{M}}^{-1}\text{q}\right|}^{2}}{1+{\text{q}}^{†}{\text{M}}^{-1}\text{q}}\right\}$$

In Equation (3) $\text{S}=\sum_{k=1}^{K}z_{k}z_{k}^{†},$ and $\text{θ}\in {\mathbb{R}}^{(2+2N+N^{2})\times 1}$ is the parameter vector, *i.e.*,

(4) $$\text{θ}={\left[\alpha_{r}\alpha_{i}{\text{q}}_{r}^{T}{\text{q}}_{i}^{T}\text{f}{\left(\text{M}\right)}^{T}\right]}^{T}={\left[{\text{θ}}_{A}^{T}{\text{θ}}_{B}^{T}\right]}^{T}$$

where

- *α_r_, α_i_* ∈ ℝ denote the real and imaginary part of *α*, respectively;
- *^T^* denotes transpose;
- ${\text{θ}}_{A}={\left[\alpha_{r}\alpha_{i}\right]}^{T}\in {\mathbb{R}}^{2\times 1}$ and ${\text{θ}}_{B}={\left[{\text{q}}_{r}^{T}{\text{q}}_{i}^{T}\text{f}{\left(\text{M}\right)}^{T}\right]}^{T}\in {\mathbb{R}}^{(2N+N^{2})\times 1}$; and observe that ***θ**_A_* contains the parameters of interest while ***θ**_B_* contains the nuisance parameters;
- ***f**(**M**)* ∈ ℝ*N*^2×1^ is a vector that contains in univocal way the real and the imaginary parts of the elements of ***M***.

As a preliminary step towards the derivation of the receivers, denote by ***J***(***θ***) the Fisher information matrix, which can be written as follows [15]

(5) $$\text{J}{\left(\text{θ}\right)}^{-1}={\left[\begin{array}{cc} {\text{J}}_{AA}(\text{θ}) & {\text{J}}_{AB}(\text{θ})\\ {\text{J}}_{BA}(\text{θ}) & {\text{J}}_{BB}(\text{θ}) \end{array}\right]}^{-1}=\left[\begin{array}{cc} {\text{C}}_{AA}(\text{θ}) & {\text{C}}_{AB}(\text{θ})\\ {\text{C}}_{BA}(\text{θ}) & {\text{C}}_{BB}(\text{θ}) \end{array}\right]$$

where

(6) $${\text{C}}_{AA}(\text{θ})={\left[{\text{J}}_{AA}(\text{θ})-{\text{J}}_{AB}(\text{θ}){\text{J}}_{BB}^{-1}(\text{θ}){\text{J}}_{BA}(\text{θ})\right]}^{-1}$$

## 3. Detector Designs

In this section we solve the binary hypothesis testing problem (1) by applying the Wald and the Rao test design criteria. Notice that problem (1) amounts to testing *H*_0_: *α* = 0 against the alternative that *α* is unrestricted.

### 3.1. Wald Test Design

The Wald test is the following decision rule [15]

(7) $${\hat{\text{θ}}}_{A,1}^{T}{\left[{\text{C}}_{AA}({\hat{\text{θ}}}_{1})\right]}^{-1}{\hat{\text{θ}}}_{A,1}\begin{array}{l} H_{1}\\ >\\ <\\ H_{0} \end{array}\eta_{1}$$

where ${\hat{\text{θ}}}_{1}={\left[{\hat{\text{θ}}}_{A,1}^{T}{\hat{\text{θ}}}_{B,1}^{T}\right]}^{T}$ with *θˆ_A_*_,1_ and *θˆ_B_*_,1_ the maximum likelihood estimates of *θ_A_* and *θ_B_* under *H*_1_, respectively, and *η*_1_ is the threshold to be set to achieve a predetermined probability of false alarm (*P_fa_*). In order to evaluate Equation (7), observe that [13]

(8) $${\left[{\text{C}}_{AA}(\text{θ})\right]}^{-1}={\text{J}}_{AA}(\text{θ})=2{\text{I}}_{2}\upsilon^{†}{\text{M}}^{-1}\upsilon$$

where ***I***_2_ denotes the 2-dimensional identity matrix, and that

(9) $${\hat{\text{θ}}}_{A,1}=\left[\begin{array}{c} 2ℜ\{\hat{\alpha }\}\\ 2ℑ\{\hat{\alpha }\} \end{array}\right]=\left[\begin{array}{c} 2ℜ\{\frac{\upsilon^{†}{{\text{S}}^{-1}}_{Z}}{\upsilon^{†}{{\text{S}}^{-1}}_{\upsilon }}\}\\ 2ℑ\{\frac{\upsilon^{†}{{\text{S}}^{-1}}_{Z}}{\upsilon^{†}{{\text{S}}^{-1}}_{\upsilon }}\} \end{array}\right]$$

where

(10) $$\hat{\alpha }=\frac{\upsilon^{†}{{\text{S}}^{-1}}_{z}}{\upsilon^{†}{{\text{S}}^{-1}}_{\upsilon }}$$

is the maximum likelihood estimate of *α*, while ℜ{·} and ℑ{·} denote the real and the imaginary parts of the argument, respectively. Now, it remains to replace *M*^−1^ in Equation (8) with its maximum likelihood estimate under *H*_1_ given by [14]

(11) $${\hat{M}}^{-1}=(K+1)\left[{\text{S}}_{1}^{-1}-\gamma \frac{{\text{S}}_{1}^{-1}(z-\hat{\alpha }\upsilon -\beta \upsilon ){\left(z-\hat{\alpha }\upsilon -\beta \upsilon \right)}^{†}{\text{S}}_{1}^{-1}}{1+\gamma {\left(z-\hat{\alpha }\upsilon -\beta \upsilon \right)}^{†}{\text{S}}_{1}^{-1}(z-\hat{\alpha }\upsilon -\beta \upsilon )}\right]$$

where

(12) $${\text{S}}_{1}=\text{S}+(z-\hat{\alpha }\upsilon ){\left(z-\hat{\alpha }\upsilon \right)}^{†}$$

(13) $$\beta =\frac{\upsilon^{†}{\text{S}}_{1}^{-1}(z-\hat{\alpha }\upsilon )}{\upsilon^{†}{\text{S}}_{1}^{-1}\upsilon }$$

(14) $$\gamma =\frac{1-(K+1){\left(z-\hat{\alpha }\upsilon -\beta \upsilon \right)}^{†}{\text{S}}_{1}^{-1}(z-\hat{\alpha }\upsilon -\beta \upsilon )}{K{\left(z-\hat{\alpha }\upsilon -\beta \upsilon \right)}^{†}{\text{S}}_{1}^{-1}(z-\hat{\alpha }\upsilon -\beta \upsilon )}$$

It is tedious but not difficult to show that

(15) $${\left[{\text{C}}_{AA}({\hat{\text{θ}}}_{1})\right]}^{-1}=2{\text{I}}_{2}\upsilon^{†}{\hat{\text{M}}}^{-1}\upsilon =2{\text{I}}_{2}(K+1)\upsilon^{†}{\text{S}}_{1}^{-1}\upsilon =2{\text{I}}_{2}(K+1)\upsilon^{†}{\text{S}}_{1}^{-1}\upsilon$$

Finally, by substituting Equations (9) and (15) into Equation (7) we obtain

(16) $$\frac{|z^{†}{\text{S}}^{-1}v{|}^{2}}{v^{†}{\text{S}}^{-1}v}\begin{array}{c} H_{1}\\ >\\ <\\ H_{0} \end{array}\eta_{1}$$

Notice that the left-hand side of Equation (16) is the decision statistic of the AMF [2], which can be also obtained by using the Wald test design criterion assuming ***q*** = 0 (homogeneous environment).

### 3.2. Rao Test Design

The Rao test for the problem (1) can be written as follows [15]

(17) $${\frac{\partial \ln f\left(z,Z;\text{θ},H_{1}\right)}{\partial {\text{θ}}_{A}}|}_{\text{θ}={\hat{\text{θ}}}_{0}}^{T}\left[C_{AA}({\hat{\text{θ}}}_{0})\right]{\frac{\partial \ln f\left(z,Z;\text{θ},H_{1}\right)}{\partial {\text{θ}}_{A}}|}_{\text{θ}={\hat{\text{θ}}}_{0}}\begin{array}{c} H_{1}\\ >\\ <\\ H_{0} \end{array}\eta_{2}$$

where

- *In f*(*z*, ***Z***; ***θ***, *H*_1_) is the natural logarithm of the pdf of *z* and ***Z*** under the *H*_1_ hypothesis (see Equation (3));
- *C_AA_*(*θˆ*_0_) = [***J**_AA_*(*θˆ*_0_)]*^−^*^1^, where ***J**_AA_*(***θ***) is given by Equation (8);
- ${\hat{\text{θ}}}_{0}={\left[00{\hat{\text{θ}}}_{B,0}^{T}\right]}^{T}$, with *θˆ*_*B*_,_0_ denoting in turn the maximum likelihood estimate of *θ_B_* under *H*_0_;
- *η*_2_ is the threshold to be set in order to ensure the preassigned *P_fa_*.

It is straightforward to show that

(18) $$\begin{array}{c} \frac{\partial \ln f(z,Z;\text{θ},H_{1})}{\partial {\text{θ}}_{A}} \end{array}=\left[\begin{array}{c} v^{†}{\text{M}}^{-1}(z-\alpha v)+{\left(z-\alpha v\right)}^{†}{\text{M}}^{-1}v\\ -jv^{†}{\text{M}}^{-1}(z-\alpha v)+j{\left(z-\alpha v\right)}^{†}{\text{M}}^{-1}v \end{array}\right]$$

(19) $$=\left[\begin{array}{l} 2ℜ\{v^{†}{\text{M}}^{-1}(z-\alpha v)\}\\ 2ℑ\{v^{†}{\text{M}}^{-1}(z-\alpha v)\} \end{array}\right]$$

where *j* denotes the imaginary unit. The maximum likelihood estimate of ***M*** under the *H_0_* hypothesis is given by [14]

(20) $$\hat{M}=\frac{1}{K+1}[{\text{S}}_{1}+\gamma (z-\xi v){\left(z-\xi v\right)}^{†}]$$

where

(21) $$\begin{array}{ll} {\text{S}}_{1}=\text{S}+zz^{†}, & \xi =\frac{v^{†}{\text{S}}_{1}^{-1}z}{v^{†}{\text{S}}_{1}^{-1}v} \end{array}$$

(22) $$\gamma =\frac{1-(K+1){\left(z-\xi v\right)}^{†}{\text{S}}_{1}^{-1}(z-\xi v)}{K{\left(z-\xi v\right)}^{†}{\text{S}}_{1}^{-1}(z-\xi v)}$$

It follows that

(23) $${\frac{\partial \ln f(z,Z;\text{θ},H_{1})}{\partial {\text{θ}}_{A}}|}_{\text{θ}=\hat{{\text{θ}}_{0}}}=\left[\begin{array}{l} 2ℜ\{v^{†}{\hat{\text{M}}}^{-1}z\}\\ 2ℑ\{v^{†}{\hat{\text{M}}}^{-1}z\} \end{array}\right]$$

(24) $$=(K+1)\left[\begin{array}{l} 2ℜ\left\{\frac{v^{†}{\text{S}}^{-1}z}{1+z^{†}{\text{S}}^{-1}z}\right\}\\ 2ℑ\left\{\frac{v^{†}{\text{S}}^{-1}z}{1+z^{†}{\text{S}}^{-1}z}\right\} \end{array}\right]$$

and

(25) $$\begin{array}{ll} {\left[{\text{J}}_{AA}({\hat{\text{θ}}}_{0})\right]}^{-1} & =2{\text{I}}_{2}\frac{1}{v^{†}{\hat{\text{M}}}^{-1}v}\\ & =2{\text{I}}_{2}\frac{1}{(K+1)\left(v^{†}{\text{S}}^{-1}v-\frac{|v^{†}{\text{S}}^{-1}z{|}^{2}}{1+z^{†}{\text{S}}^{-1}z}\right)} \end{array}$$

Gathering the above results, the Rao test can be recast as

(26) $$\frac{|v^{†}{\text{S}}^{-1}z{|}^{2}}{v^{†}{\text{S}}^{-1}v(1+z^{†}{\text{S}}^{-1}z)}\left[\frac{1}{1+z^{†}{\text{S}}^{-1}z-\frac{|v^{†}{\text{S}}^{-1}z{|}^{2}}{v^{†}{\text{S}}^{-1}v}}\right]\begin{array}{l} H_{1}\\ >\\ <\\ H_{0} \end{array}\eta_{2}$$

or, equivalently, as

(27) $$\frac{|v^{†}{\left(\text{S}+zz^{†}\right)}^{-1}z{|}^{2}}{v^{†}{\left(\text{S}+zz^{†}\right)}^{-1}v}\begin{array}{l} H_{1}\\ >\\ <\\ H_{0} \end{array}\eta_{2}$$

which is the H-RAO [4].

## 4. Conclusions

This work addresses the adaptive detection of point-like targets in nonhomogeneous scenarios. In particular, at the design stage it is assumed that the CUT contains a noise-like interferer in addition to thermal noise, clutter, and to the possible signal of interest; a set of secondary data, free of signal components, is available: such data share a common covariance matrix that is equal to that of thermal noise plus clutter in the cell under test. Observe that the covariance mismatch between the CUT and the secondary data can lead to receivers that are less inclined to reveal a signal with the actual steering vector different from the nominal one (selective receivers). In addition, we assume that the noise-like interferer is orthogonal to the nominal steering vector in the whitened observation space. Under the above assumptions, we show that the Wald test coincides with the AMF, while the RAO test is the H-RAO. The latter result provides an alternative explanation of the good selectivity properties exhibited by the H-RAO [5]. Moreover, observe that the condition (2) makes the H-RAO less selective than the DN-AMF, derived in [13] using the Rao test design criterion without any constraint on the noise-like interferer.

As a final comment, it would be of interest to investigate under which conditions these design criteria are invariant with respect to different classes of detection problems. A preliminary step towards this direction is given in [16].
